# Growths of SiC Single Crystals Using the Physical Vapor Transport Method with Crushed CVD-SiC Blocks Under High Vertical Temperature Gradients

**DOI:** 10.3390/ma17235789

**Published:** 2024-11-26

**Authors:** Ju-Hyeong Sun, Jae-Hyeon Park, Si-Young Bae, Yun-Ji Shin, Yong-Jin Kwon, Won-Jae Lee, Se-Hun Kwon, Seong-Min Jeong

**Affiliations:** 1 Semiconductor Materials Center, Korea Institute of Ceramic Engineering and Technology (KICET), Jinju 52851, Republic of Korea; ssun630@kicet.re.kr (J.-H.S.); 1q2w3e44r@naver.com (J.-H.P.); shinyj@kicet.re.kr (Y.-J.S.); 2 School of Materials Science and Engineering, Pusan National University, Busan 46241, Republic of Korea; 3 Major of Semiconductor Engineering, Pukyong National University, Busan 49315, Republic of Korea; siyoungbae@pknu.ac.kr; 4 Hana Materials Inc., Asan 31413, Republic of Korea; yjkwon@hanamts.com; 5 School of Advanced Materials Engineering, Dong-Eui University, Busan 51140, Republic of Korea; leewj@deu.ac.kr

**Keywords:** SiC, single crystal, crystal growth, PVT, CVD-SiC, rapid growth

## Abstract

A recent study reported the rapid growth of SiC single crystals of ~1.5 mm/h using high-purity SiC sources obtained by recycling CVD-SiC blocks used as materials in semiconductor processes. This method has gained attention as a way to improve the productivity of the physical vapor transport (PVT) method, widely used for manufacturing single crystal substrates for power semiconductors. When recycling CVD-SiC blocks by crushing them for use as sources for growing SiC single crystals, the properties and the particle size distribution of the material differ from those of conventional commercial SiC powders, making it necessary to study their effects. Therefore, in this study, SiC single crystals were grown using the PVT method with crushed CVD-SiC blocks of various sizes as the source material, and the growth behavior was analyzed. Simulation results of the temperature distribution in the PVT system confirmed that using large, crushed blocks as the SiC source material generates a greater temperature gradient within the source compared to conventional commercial SiC powder, making it advantageous for rapid growth processes. Additionally, when the large, crushed blocks were vertically aligned, good crystal quality was experimentally achieved at high growth rates, even under non-optimized growth conditions.

## 1. Introduction

Silicon carbide (SiC) single-crystal substrates have been increasingly adopted for semiconductor devices in high-power and high-frequency applications due to their superior thermal and electrical properties compared to conventional silicon substrates [1,2]. SiC single crystals are grown as high-quality bulk crystals for semiconductor applications using methods such as the physical vapor transport (PVT) method, the High-Temperature Chemical Vapor Deposition (HTCVD) method, and the Top-Seeded Solution Growth (TSSG) method. Among these, the PVT method, which was the first to be commercialized, is now widely used as a mass production process.

The PVT method involves sublimating a solid source material at high temperatures and then physically transporting the sublimated vapor species to a seed crystal, where the crystal growth occurs. Although the development of SiC single crystal growth using the PVT method has a long history, achieving high-quality single crystals in actual manufacturing processes remains a challenging task. SiC crystal growth typically occurs in a sealed graphite crucible heated to over 2000 °C using induction heating, and the process continues for an extended period. Because real-time monitoring of the system during the process is nearly impossible from the outside, optimizing process parameters is extremely challenging and requires long-term research and development through a trial-and-error approach [3].

Recently, studies have reported on the growth of SiC single crystals using the PVT method with high-purity SiC source materials obtained by recycling CVD-SiC blocks used in semiconductor processes [4,5]. Although the PVT growth method remains the same, using crushed CVD-SiC blocks instead of conventional synthetic powders introduces significant differences in the material properties and particle size distribution of the source material, which can greatly influence the process variables. However, manufacturers already utilize well-established processes for growing SiC single crystals based on decades of research and development using conventional source materials. Therefore, developing a PVT process that applies crushed CVD-SiC blocks as a new source material suggests the need to redefine the well-established PVT growth process for SiC single crystals.

Although many studies have reported on the effects of various process variables in the growth of SiC single crystals using the PVT method, research on the influence of the source material remains very limited [6,7]. Recently, Jeong et al. reported that applying CVD-SiC blocks allowed them to grow SiC single crystals with excellent crystal quality at a high rate of 1.46 mm/h using the PVT method [5]. The ability to rapidly grow SiC single crystals using the PVT method suggests a potential for revolutionary improvements in the manufacturing time and cost of SiC single crystal substrates. This has increased interest in using CVD-SiC as new source material.

In this study, we aimed to develop the PVT process for SiC single crystal growth using CVD-SiC source material by employing crushed CVD-SiC blocks of various sizes. We designed the hot zone structure through simulations and applied size-classified crushed CVD-SiC blocks as the source material. We then analyzed the changes in the source material before and after growth, as well as the characteristics of the grown single crystals. By comparing the simulation results with the experimental growth outcomes, we investigated the mass transport mechanism within the source material and the crystal growth behavior using the crushed CVD-SiC blocks in the PVT method.

## 2. Modeling and Experimental

**Figure 1:**
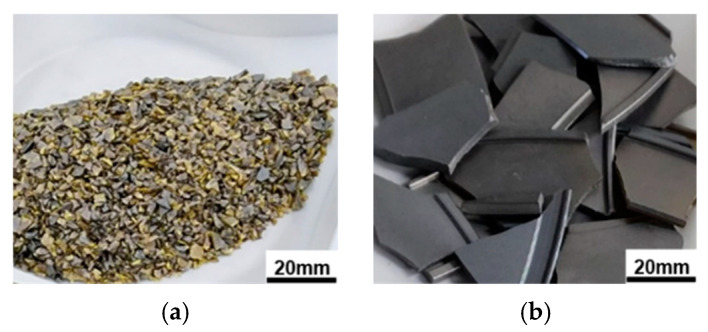
Appearance of crushed CVD-SiC block sources: (**a**) small size (S1) and (**b**) large size (S2).

In this study, 4H-SiC single crystals were grown using the PVT method with crushed high-purity SiC polycrystalline blocks synthesized via the CVD process using Methyltrichlorosilane (MTS, CH_3_Cl_3_Si) as the precursor. CVD-SiC blocks are grown on graphite susceptors of 300 mm diameter and 10 mm thickness, crushed to the required particle size through a jaw crusher with tungsten jaws, washed with DI water, and dried to prepare the SiC raw material used in this study. The color of the source material varies depending on factors such as particle size, polytype, doping level, and crystal orientation [4]. As shown in Figure 1, the S1 sample, which has the smallest particle size, exhibited a mixture of black and yellow colors, whereas no significant color variation was observed in the larger particle-sized S2 sample. The crushed CVD-SiC block sources were analyzed using an optical microscope (VHX-7000, Keyence Corporation, Osaka, Japan), particle size analyzer (LA-960, Horiba Ltd., Kyoto, Japan), and High-Resolution X-ray Diffraction (HR-XRD, SmartLab-XRD, Rigaku Holdings Corporation, Tokyo, Japan). The purities of the crushed CVD-SiC block sources were measured with glow discharge mass spectrometry (GDMS) in an independent laboratory (Eurofins EAG Materials Science, Columbia, SC, USA).

**Figure 2:**
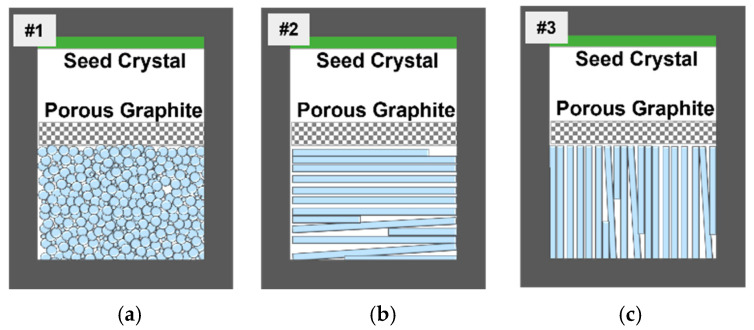
Schematics of the alignment configurations of crushed CVD-SiC block sources for PVT growth under (**a**) condition #1, (**b**) condition #2 and (**c**) condition #3.

**Figure 3:**
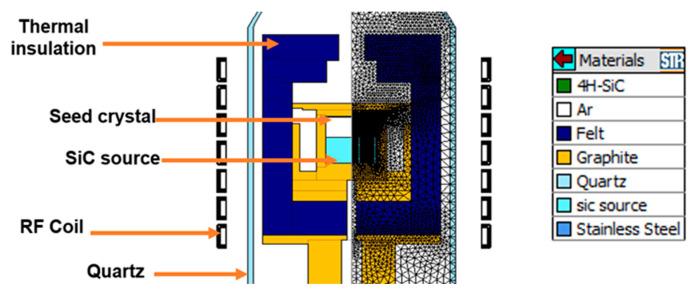
Drawing of the hot zone for PVT growth in the induction heating furnace and its meshed model.

Figure 2 presents a schematic diagram of the hot zone used in the PVT process with S1 and S2. For the S2 sample, considering the plate-like shape, we evaluated the SiC single crystal growth behavior by arranging the sample in two different alignments: horizontally stacked and vertically aligned. To address the issue of uneven gas supply to the growth surface through channels formed between the large-sized particles, a porous graphite (PG) plate (PG-70, Morgan Advanced Materials plc, Windsor, UK) was placed above the source as a diffusion layer. Initially, we planned to use as thick a plate as possible in anticipation of the diffusion effect within the PG plate. If the PG plate is too thick, however, there was a problem that the grown crystal surface touches the PG plate when growing for a long time, so we adopted the 1.5 mm-thick PG plate. A seed crystal with a 50 mm diameter, n-type, 4° off-axis, 4H-SiC, Carbon face was used. The growth process was designed using the commercial simulation code VR-PVT 8.2 (STR, Belgrade, Republic of Serbia) to ensure high growth rates under a steep vertical temperature gradient [8]. Figure 3 shows the 2D axisymmetric model and its meshed model used for the simulation using VR-PVT. A copper coil was modeled around the quartz tube as in the real reactor, and an inner graphite crucible containing raw materials heated by thermal radiation from an outer graphite crucible with induction heating was placed. The gas part of the model, which requires heat and mass transfer analysis, is composed of fine elements, especially for fluid analysis. The material properties and process parameters used in the simulation were based on values from the literature [5]. Si melting test was performed using the reactor to calibrate the pyrometers’ emissivity values so that the pyrometers accurately read the melting point of Si (1414 °C), and then the temperatures of the reactor were measured using the calibrated pyrometers. The single crystal growth was carried out for 4 h at a pressure of ~4 kPa in an Ar gas atmosphere with the bottom temperature of the crucible set at ~2350 °C. Table 1 summarizes the growth conditions used in this study.

After crystal growth, the grown crystals were analyzed using micro X-ray computed tomography (micro-CT, Inspexio SMX-225XT, Shimadzu, Kyoto, Japan) to evaluate the cross-sectional morphology under different experimental conditions. Additionally, Raman spectroscopy (UniDRON Raman spectroscopy, UniNanoTech, Yongin, Republic of Korea) with a 532 nm wavelength was used for polytype and stacking fault analysis. High-resolution X-ray diffraction (HR-XRD, SmartLab-XRD, Rigaku Holdings Corporation, Tokyo, Japan) was conducted to analyze the crystal quality with the Full Width at Half Maximum (FWHM).

## 3. Results and Discussion

### 3.1. Preparation of Source Materials for PVT Growth

During the PVT process, the mass transport of the sublimed vapor species is carried out in two stages: first, the transport of sublimated vapor species through the porous medium between the source particles, and second, the transport of these vapor species from the top of the source material to the crystal growth front through atmospheric space. In many industrial cases, the process design of the PVT method has been based on the material transport mechanism between the top of the source and the seed crystal surface. In this stage, the movement of vapor species in free space is driven by the temperature difference between the top of the source material and the seed crystal surface and is primarily governed by pressure [9,10]. In contrast, understanding the material transport through the source particles has received less attention in the current SiC mass production processes using well-defined source materials, resulting in fewer studies in this area [11,12,13].

**Figure 4:**
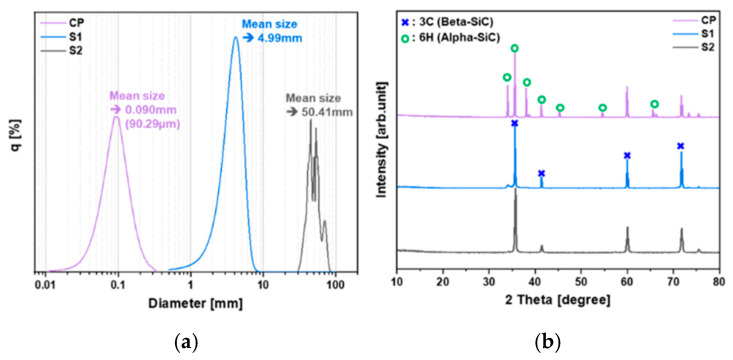
(**a**) Particle size distribution of crushed CVD-SiC block sources (S1, S2) and commercial powder (CP). (**b**) X-ray diffraction patterns for S1, S2, and CP.

To the authors’ knowledge, typical commercial SiC powders for SiC growth typically have an average particle diameter of 50 to 500 μm. Of course, larger particles are preferred because they generate less dust during the growth process and are more pure. However, the source used in this study has a very large particle size, with an average particle diameter of over 4.9 mm. Therefore, it was predicted that the current PVT process employing ultra-large sources would have a different temperature distribution and material migration within the source material than the conventional SiC growth process employing commercial powder sources. The crushed CVD-SiC blocks used in this study were so large that it was difficult to obtain results using the wet particle size analysis method commonly employed for source material analysis. Therefore, the particle size was obtained from the average value for 10 samples for each condition using an optical microscope, and the results were presented in Figure 4a, along with the wet particle size analysis data for the commercial powder (CP) used as a comparison. The analysis revealed that the mean size of the smallest S1 particles was approximately 5 mm, while the largest particles had a mean size of about 50 mm. The particle size of the crushed CVD-SiC block source material was over 500 times larger than that of the commercial powder (CP), which had a mean size of ~90 μm. As a result, the heat transfer characteristics within the crushed CVD-SiC blocks are expected to differ significantly from those within the commercial powder. The crushed CVD-SiC blocks had a plate-like shape, unlike the spherical commercial powder (CP), with a thickness of about 1 mm. Due to this plate-like shape, the paths through which the sublimated vapor species moved would vary depending on the spatial arrangement of the source material, potentially causing significant variations in material transport based on the source alignment.

Since the CVD-SiC blocks were synthesized via a CVD process at temperatures above 1300 °C, they were expected to be more advantageous for process control due to their more uniform 3C-SiC crystalline phase, compared to the commercial powder (CP), which typically contains a mixed phase of 3C-SiC and 4H/6H-SiC, as confirmed by the XRD results in Figure 4b. Although the vapor species of 3C-SiC, a beta phase, might have a slightly higher Si/C ratio compared to the alpha phase 4H/6H-SiC [14,15,16], the significantly larger particle size of the crushed CVD-SiC block source suggests that the thermodynamic contribution of surface energy would be minimal, leading to sublimation at higher temperatures. In the PVT growth process of SiC, the sublimation temperature of carbon is thermodynamically higher than that of silicon, which is experimentally confirmed by the residual carbonized SiC powder after growth. The fact that the sublimation temperature of carbon is higher than that of silicon thermodynamically means that as the temperature increases beyond the sublimation temperature of silicon, the sublimation of carbon also increases, leading to a decrease in the Si/C ratio of the sublimated vapor species. Therefore, when sublimation occurs at higher temperatures, the reduced Si/C ratio of the sublimated vapor species can offset the effects associated with the difference in polytypes mentioned above. As a result, the actual Si/C ratio contributing to the growth system may not differ significantly between the crushed CVD-SiC block source and the commercial powder.

Table 2 shows the purity of the source materials analyzed using GDMS. While trace amounts of aluminum, boron, and phosphorus, which influence doping, were detected in all forms of the source samples, the impurity contents of all crushed CVD-SiC blocks were generally lower than those of the commercial powder. However, metal impurities such as iron and tungsten were found to be relatively higher in the smaller particle-sized S1 sample, likely due to the introduction of metal impurities during the crushing process with tungsten jaws of the CVD-SiC blocks into smaller particles. In addition, powders composed of small particles with a large specific surface area generally have lower impurity concentrations than powders composed of large particles with a small specific surface area [17]. The GDMS analysis results indicated that the purity of the crushed CVD-SiC block source materials ranged from 99.99877% (4N8) to 99.99966% (5N6), which is higher than the 99.99530% (4N5) purity level of the commercial products.

### 3.2. Temperature Distribution Dependence on Particle Size Analyzed Through Simulations

**Figure 5:**
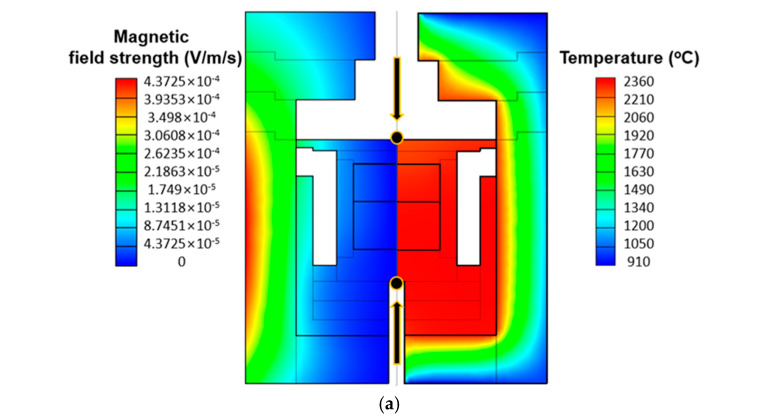

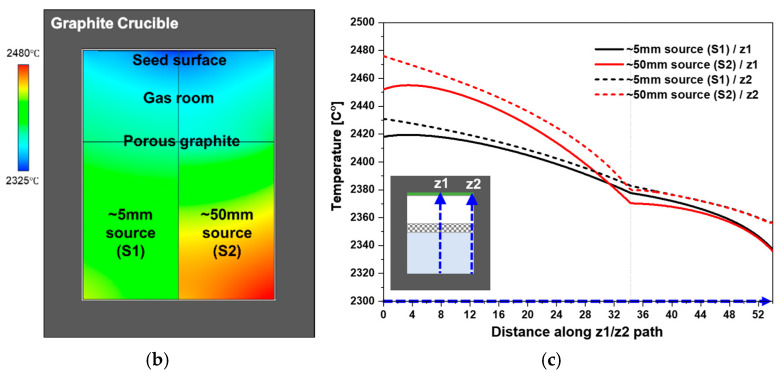
(**a**) Simulation results of magnetic field strength and temperature distribution for S1 case. (**b**) Simulation results of temperature distribution depending on the particle size. (**c**) Temperature distribution along paths of z1 and z2.

The simulation results of the electromagnetic field and temperature distribution for the S1 sample are shown in Figure 5a. As shown in Table 3, the simulation results were verified by comparing the temperature distribution with the experimentally obtained temperature distribution for the S1 sample, and the simulation accuracy was estimated to be 95.6% based on the temperature difference between the top and bottom of the crucible.

As shown in the electromagnetic field distribution and temperature distribution in Figure 5a, in the induction heating system with hot zone configuration used in this study, the electromagnetic field is estimated to be strong on the outer crucible close to the induction coil, causing joule heat due to the induction current, which is particularly strong in the outer crucible part, the generated heat is estimated to be effectively transferred to the inner crucible by the heat transfer mechanism including thermal radiation.

To investigate the differences in heat transfer behavior based on particle size, PVT simulations were conducted considering the S1 sample with a smaller particle size and the S2 sample with a larger particle size. The VR-PVT simulation package used in this study does not account for the specific transport paths of sublimated vapor species when coarse source materials are employed, so the simulations focused solely on analyzing the temperature distribution within the PVT hot zone under steady-state conditions, assuming the initial growth phase. As shown in Figure 5b, the overall temperature gradient was relatively larger when using the larger-sized source material, S1, compared to the smaller-sized material, S2. The temperature of the seed crystal was estimated to result in a convex-shaped crystal growth, with the crucible wall being hotter and the center of the crucible cooler [18]. The horizontal temperature difference on the seed crystal was approximately 20 °C, regardless of the particle size of the source material.

To further analyze the temperature distribution dependence on particle size in both vertical and horizontal directions, the temperature distribution along the vertical (*z*-axis) and horizontal (*x*-axis) paths was evaluated. As shown in Figure 5c, the paths from the crucible bottom to the lid were defined as z1 along the centerline of the crucible and z2 along the crucible wall, and the temperature distribution along these paths was plotted. Along both paths, a typical temperature distribution characteristic of the PVT method was observed, with the bottom of the graphite crucible being hotter and the lower part of the lid being cooler. The temperature along the z2 path, which represents the crucible wall in contact with the inductively heated graphite crucible, was generally higher than that along the z1 path along the centerline of the crucible. The vertical temperature difference from the bottom of the crucible to the lower part of the lid was 116.1 °C when using the larger S2 source material and 80.9 °C when using the smaller S1 source material, indicating that the vertical temperature gradient was approximately 1.4 times higher with the larger source material. Especially in the source region, the vertical temperature gradient was 81.8 °C for the S2 material and 40.3 °C for the S1 material, showing that the vertical temperature gradient was about twice as high in the larger source material. Similarly, the vertical temperature difference along the z2 path was 119 °C for the S2 material and 73.5 °C for the S1 material, with the larger source material exhibiting a vertical temperature gradient approximately 1.6 times higher, resulting in a greater vertical temperature gradient than along the z1 path. The vertical temperature difference within the source material along the z2 path was also found to be similar to that along the z1 path.

The reason for this difference in temperature gradients is attributed to the fact that, as reported in some literature, larger source particles have a lower surface area-to-volume ratio, reducing radiative heat transfer [19]. Additionally, larger particles have greater mass at the same density, increasing their heat capacity, which means that more heat is required for a single particle to reach the same temperature [20]. Therefore, when using larger source particles in the PVT process, it may be necessary to supply more heat compared to using smaller source particles. These factors contribute to the significant temperature differences between the source material adjacent to the heated crucible and the material in the central region when using larger particles in the PVT process, as confirmed by the temperature distributions of S1 and S2 shown in Figure 5a, which indicate the presence of a large temperature gradient within the source material. The simulation results showed that the horizontal temperature gradient at the bottom of the crucible was 23.5 °C for the S2 source material and 12 °C for the S1 source material, which is likely due to the lower heat transfer efficiency of the larger source particles mentioned above.

The CVD-SiC blocks used in this study can have their particle size adjusted through controlled crushing processes. When CVD-SiC blocks with larger particle sizes are used as the source material in the PVT process, they generate larger vertical and horizontal temperature gradients compared to conventional PVT processes that use commercial powders. This generates a strong driving force for the mass transport of sublimated vapor species, which is advantageous for increasing the crystal growth rate.

### 3.3. Investigation of the Remaining Sources and the Grown Crystals After PVT Growth

**Figure 6:**
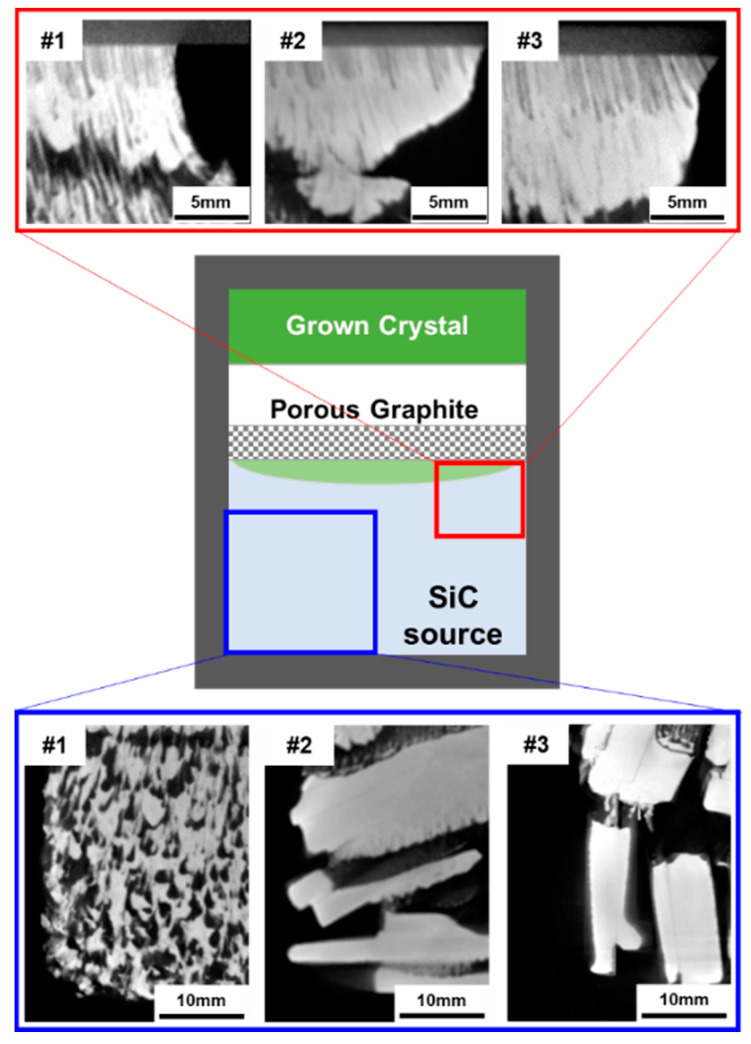
X-ray CT images of remaining CVD-SiC block sources after PVT growth in the source regions.

Figure 6 shows cross-sectional X-ray computed tomography (X-ray CT) images of the remaining CVD-SiC block sources after PVT growth. The sublimed source region is highlighted within the blue square, and the region near the PG plate is highlighted within the red square. During the growth process, the SiC source begins to sublimate and carbonize from the lower outer part of the crucible, where temperatures are higher. As growth progresses, the inner portions of the source material are gradually consumed. When using conventional SiC powder as the source material, the path of sublimated vapor species typically develops into a series of micro-channels within the porous medium of the source powder [21]. These micro-channels develop further during the growth process as vapor species, supplied by smaller particles that heat up and sublimate at lower temperatures, condense on the surface of larger particles that heat up more slowly [13,21,22,23].

In this study, using crushed CVD-SiC block sources, a similar development of micro-channels was observed in the source region of experiment #1, where the smallest particle size (S1) was used. This is similar to the micro-channel formation observed in PVT growth using commercial powder sources. However, in experiments #2 and #3, where the larger particle size (S2) was used, such micro-channels were hardly observed. When using plate-like crushed CVD-SiC blocks, the transport of vapor species appears to follow macro-channels formed between the plate-like blocks, determined by the shape and arrangement of the crushed blocks. These results indicate that as particle size increases, the transport path of vapor species within the source material shifts from micro-channels to macro-channels.

Experiments #2 and #3 were identical, with the only difference being the arrangement of the crushed blocks, allowing for the evaluation of the effect of macro-channel directionality formed by the block arrangement. This comparative experiment could be generalized as a comparison between macro-channels that are parallel to the temperature gradient, which is the driving force of the mass transport of sublimed vapor species, and those that are perpendicular to it. When the macro-channels are parallel to the temperature gradient, the vapor species can be rapidly transported. However, when the macro-channels are perpendicular, the vapor species must pass through gaps between the crushed blocks along vertical directions, increasing the distance and reducing the speed of transport. For this reason, in experiment #3, the vapor species pass through the source material more quickly compared to experiment #2, resulting in differences in growth rates. In this study, a low-temperature region formed beneath the PG plate, leading to recrystallization.

It was observed that experiment #3, with shorter and faster transport paths, resulted in a larger recrystallized area compared to experiment #2, as shown in Figure 6. The PG plate was intentionally placed above the source material to improve the uniformity of material supply, which could be uneven due to the use of large, crushed SiC blocks. The recrystallization beneath the PG plate suggests that, instead of functioning as a diffusion layer for uniform material supply, the PG plate acted more as a heat sink due to its large surface area. This caused cooling and recrystallization of the sublimated material before it could reach the seed crystal. This recrystallization at the cooled PG plate is inefficient, as it consumes some of the sublimated material, ultimately reducing the yield of single crystals and leading to a lower growth rate than previously reported in similar studies [5]. These findings underscore the need for further optimization of the hot zone structure when using crushed CVD-SiC block sources in the PVT process to develop an optimal growth process for SiC single crystals.

**Figure 7:**
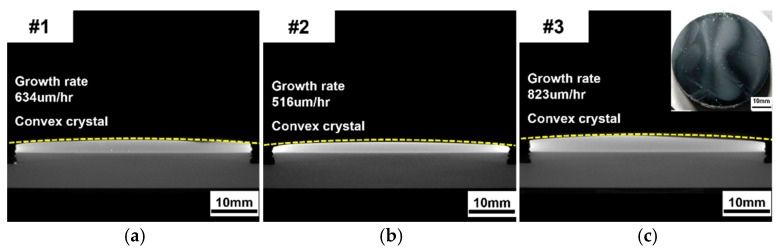
X-ray CT images of grown SiC crystals using sources (**a**) #1, (**b**) #2, and (**c**) #3.

Figure 7 shows cross-sectional X-ray computed tomography (X-ray CT) images of SiC crystals grown from crushed CVD-SiC block sources. None of the grown crystals exhibited microcracks, inclusions, or polycrystalline regions. Despite the significant consumption of vapor species due to recrystallization beneath the PG plate, the vertical temperature gradient increased with larger particle sizes, leading to a tendency for higher growth rates. As shown in Figure 7, the crystals displayed convex shapes with surface curvatures of 2.31 m^−1^, 3.02 m^−1^, and 3.73 m^−1^. As the particle size of the source increased, higher surface curvatures were observed. According to the simulation results shown in Figure 5, the horizontal temperature gradient on the top of the PG plate significantly increased with larger particle sizes, while the horizontal temperature gradient on the seed crystal did not show noticeable changes depending on the particle size. Since the temperature gradient represents the driving force of vapor transport in the PVT system, the simulation results indicate that vapor transport is directed from the outer region to the central region at the top of the PG plate, which may explain the higher growth rate at the center of the seed crystal, despite the recrystallization occurring beneath the PG plate.

**Figure 8:**
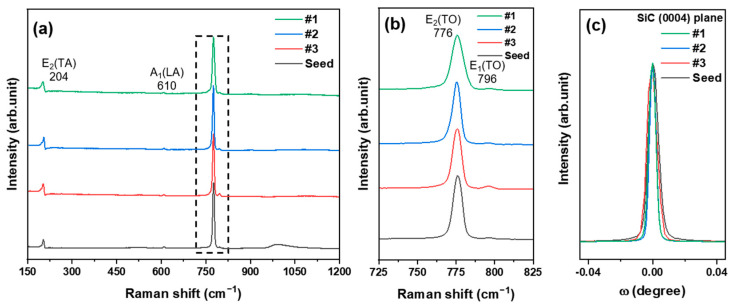
Polytype and crystallinity analysis of growth crystals: (**a**) Raman spectra of the grown crystals from source #1, #2, #3, and CP, (**b**) FTO peaks of the Raman spectra, (**c**) X-ray rocking curves for the grown crystals from source #1, #2, #3, and CP.

Figure 8 shows the Raman spectra and X-ray diffraction ω-rocking curves (XRC) for the grown crystals and the seed crystal. The Raman spectra of the grown crystals, using crushed CVD-SiC block sources predominantly consisting of the 3C-SiC phase, revealed that all growth conditions produced single-phase 4H-SiC crystals, as indicated by the transverse acoustic (TA) peak at 204 cm^−1^ and the transverse optical (TO) peaks at 776 and 796 cm^−1^ (Figure 8a). The absence of a significant longitudinal optical (LO) peak at 964 cm^−1^ in all conditions suggests that the crushed CVD-SiC block source material was already doped with nitrogen [24,25].

When the growth rate of SiC crystals is high, the step-flow growth mechanism through the expansion of atomic-level terraces present on the surface of the seed substrate is destabilized, which is prone to step bunching during growth, which in turn leads to the phenomenon of increasing stacking disorder in the grown crystals [26].

To analyze the stacking defects in the grown crystals at high growth speed, the TO peak at 796 cm^−1^ [27,28], which is associated with stacking defects in 4H-SiC from Raman analysis, was analyzed by calculating the peak-to-peak ratio for the main TO peak at 776 cm^−1^. These ratios were 0.201 (#1), 0.088 (#2), 0.147 (#3), and 0.075 (seed), which interestingly indicated that the rapidly grown crystal (#3) had a relatively low level of stacking defects relative to its growth rate. The XRC results shown in Figure 8c revealed that the FWHM values of each crystal were 18.3 arcsec (#1), 16.6 arcsec (#2), 26.5 arcsec (#3), and 26.5 arcsec (seed). These values are comparable with recent commercial SiC wafers’ values under 30 arcsec [29,30,31], which indicates good crystallinity despite the high growth rates in this study.

This study demonstrates that large temperature gradients can enable the rapid growth of SiC single crystals when using coarse source materials and that optimizing the design of macro-channels within the source material can facilitate high-speed growth. To further stabilize the PVT process using coarse materials, additional process optimization distinct from conventional processes using commercial powders will be necessary.

Various further studies are required to apply the results of this study, which were demonstrated by growing 2-inch SiC single crystals, to commercial large-area SiC substrate fabrication processes. In particular, follow-up studies on the design and configuration of the PG plate, which was demonstrated to have a significant effect on the temperature gradient in the reactor in this study, are expected to contribute to the optimization of the rapid growth of SiC crystals with extra-large sources [32].

## 4. Conclusions

In this study, SiC single crystals were grown using the PVT method with crushed CVD-SiC blocks of various sizes under a high vertical temperature gradient. The crystal growth behavior when using large particle-sized sources was investigated. We found that the PG plate on the source significantly influenced the temperature of the source surface, which could lead to the recrystallization of the source vapor, blocking the efficient use of the source material. This study also confirmed that the size, shape, and arrangement of crushed CVD-SiC sources have a significant impact on the temperature gradient and mass transport characteristics during SiC growth using the PVT method. It was interesting to note that good crystal quality was achieved at high growth rates when large, crushed blocks were vertically aligned, forming macro-channels of the vaporized source.

This study opens up many research topics related to the rapid growth of SiC crystals based on extra-large particle sources, suggesting the proper adoption of PG plates, a systematic study of macro-channels, and dislocation propagation under rapid growth conditions. These topics should be explored further to apply rapid growth techniques to the mass production of SiC substrates.

## Figures and Tables

**Table 1:** Conditions for PVT growth using crushed CVD-SiC block sources with various alignments.

No.	Source	Source Alignment	Layout Schematic	Growth Condition				
				Top (°C)	Bottom (°C)	Time (h)	Pressure (kPa)	Atmosphere
#1	S1+PG	Random	Figure 2a	2244	2359	4	4	Argon
#2	S2+PG	Horizontal	Figure 2b	2249	2342			
#3	S2+PG	Vertically	Figure 2c	2249	2356			

**Table 2:** Impurities of SiC sources CP, S1, and S2, measured by GDMS.

Elements		Commercial Powder (CP) (ppm)	Crushed CVD-SiC Source (ppm)	
			S1	S2
p-type dopant	Al	25	0.2	8
	B	6	0.14	0.19
n-type dopant	P	0.18	<0.05	0.3
Other (Fe, W, Ti, etc.)		15.83	10.07	3.58
Total		47.01	10.41	12.34
Purity (%)		99.99530	99.99896	99.99877

**Table 3:** Calculation of simulation Accuracy for model validation.

Crucible Top		Crucible Bottom		Temperature Difference		Accuracy *
Exp.(T1)	Sim.(T2)	Exp.(T3)	Sim.(T4)	Exp.(T3-T1)	Sim.(T4-T2)	
2244 °C	2245 °C	2359 °C	2355 °C	115 °C	110 °C	95.6%

* Accuracy (%) = 100 − Simulation Error (%) = (1 − |(1 − (T4 − T2)/(T3 − T1)|) × 100.

## Data Availability

The data presented in this study are openly available in Open Science Framework at https://doi.org/10.17605/OSF.IO/5ZPAB.
